# SMURF1 facilitates estrogen receptor ɑ signaling in breast cancer cells

**DOI:** 10.1186/s13046-018-0672-z

**Published:** 2018-02-12

**Authors:** Huijie Yang, Na Yu, Juntao Xu, Xiaosheng Ding, Wei Deng, Guojin Wu, Xin Li, Yingxiang Hou, Zhenhua Liu, Yan Zhao, Min Xue, Sifan Yu, Beibei Wang, Xiumin Li, Gang Niu, Hui Wang, Jian Zhu, Ting Zhuang

**Affiliations:** 1 Henan Key Laboratory of immunology and targeted therapy, School of Laboratory Medicine, Henan Collaborative Innovation Center of Molecular Diagnosis and Laboratory Medicine, Xinxiang Medical University, Xinxiang, 453003 Henan Province People’s Republic of China; 2 Department of Gastroenterology, The Third Affiliated Hospital of Xinxiang Medical University, Xinxiang, Henan China; 3 Phil Rivers Technology (Beijing) Ltd, Beijing, China; 4 Department of Cancer genomics, LemonData biotech (Shenzhen) Ltd, Shenzhen, China; 5 Department of Medical Oncology, Peking University International Hospital, Beijing, China; 6 Department of General Surgery, Beijing Friendship Hospital, Capital Medical University, National Clinical Research Center of Digestive Diseases, Beijing Key Laboratory of Cancer Invasion and Metastasis Research & National Clinical Research Center of Digestive Diseases, Beijing, 100050 China; 7 Department of Physiology, University of Texas Southwestern Medical Center, Dallas, TX 75390 USA; 8 Key Laboratory of Carcinogenesis and Translational Research (Ministry of Education) Department of Renal cancer and Melanoma, Peking University School of Oncology, Beijing Cancer Hospital and Institute, Beijing, China; 9 Center for Cancer Research, Xinxiang Medical University, Xinxiang, Henan China; 10 Department of Molecular Biology, University of Texas Southwestern Medical Center, Dallas, TX 75390 USA; 11 Xinxiang Medical University, School of Laboratory Medicine, Xinxiang, Henan Province China

**Keywords:** SMURF1, ER alpha, Breast cancer, Ubiquitination, Protein stability

## Abstract

**Background:**

Estrogen receptor alpha (ER alpha) is expressed in the majority of breast cancers and promotes estrogen-dependent cancer progression. ER alpha positive breast cancer can be well controlled by ER alpha modulators, such as tamoxifen. However, tamoxifen resistance is commonly observed by altered ER alpha signaling. Thus, further understanding of the molecular mechanisms, which regulates ER alpha signaling, is important to improve breast cancer therapy.

**Methods:**

SMURF1 and ER alpha protein expression levels were measured by western blot, while the ER alpha target genes were measured by real-time PCR. WST-1 assay was used to measure cell viability; the xeno-graft tumor model were used for in vivo study. RNA sequencing was analyzed by Ingenuity Pathway Analysis. Identification of ER alpha signaling was accomplished with luciferase assays, real-time RT-PCR and Western blotting. Protein stability assay and ubiquitin assay was used to detect ER alpha protein degradation. Immuno-precipitation based assays were used to detect the interaction domain between ER alpha and SMURF1. The ubiquitin-based Immuno-precipitation based assays were used to detect the specific ubiquitination manner happened on ER alpha.

**Results:**

Here, we identify the E3 ligase SMURF1 facilitates ER alpha signaling. We show that depletion SMURF1 decreases ER alpha positive cell proliferation in vitro and in vivo. SMURF1 depletion based RNA-sequence data shows SMURF1 is necessary for ER alpha target gene expression in the transcriptomic scale. Immunoprecipitation indicates that SMURF1 associates with the N-terminal of ER alpha in the cytoplasm via its HECT domain. SMURF1 increases ER alpha stability, possibly by inhibiting K48-specific poly-ubiquitination process on ER alpha protein. Interestingly, SMURF1 expression could be induced via estradiol treatment.

**Conclusions:**

Our study reveals a novel positive feedback between SMURF1 and ER alpha signaling in supporting breast cancer growth. Targeting SMURF1 could be one promising strategy for ER alpha positive breast cancer treatment.

**Electronic supplementary material:**

The online version of this article (10.1186/s13046-018-0672-z) contains supplementary material, which is available to authorized users.

## Background

Breast cancer ranks number one in woman malignancy worldwide and causes the second most frequent cancer mortality in females [1]. About 60–70% breast cancers are estrogen receptor alpha (ER alpha) positive, where ER alpha transcription program is required for breast cancer progression [2]. ER alpha belongs to the ligand-dependent subfamily of the nuclear receptor or transcription factors, and its activity is mainly regulated by estrogen [3]. Like other nuclear receptors, ERα has several distinct domains: Activator Function 1 (AF1) domain at the N-terminus that recruit co-factors, DNA-binding domain (DBD) that binds to the estrogen response elements (EREs), and Activator Function 2 (AF2) domain that is the ligand-dependent transactivation domain [3]. As part of ER alpha transactivation function, the AF2 domain recruits several co-activators and co-repressors to control ERα activity [4]. Upon estrogen stimulation, the ER alpha protein can shuttle into the nuclear and bind to cis-regulatory DNA regions of target genes, which subsequently trans-activates certain gene expression and promotes cancer progression.

Since ER alpha signaling is necessary for the progression of luminal type of breast cancers, hormone depletion and ERα antagonists have been widely used to treat ER^+^ breast cancer patients, such as tamoxifen [5]. Although tamoxifen largely improves breast cancer patient survival, the development of tamoxifen resistance is common. Thus the understanding of dys-regulation of ER alpha that trigger inappropriate estrogen signaling and drug resistance is of utmost importance. A number of confirmed and possible mechanisms, which triggers inappropriate estrogen signaling, are shown to relate to the transcriptional and epigenetic control of ER alpha protein [6]. Others that account for the dys-regulation of estrogen signaling involve the controls of ER alpha activities by the ligands, cofactors and post-translational modifications [7, 8].

Although the understanding of how ER alpha protein are controlled in breast cancer is largely unclear, the regulation of ER alpha protein stability is evident from that recent studies showing that several post-translational modifications are involved in ER alpha protein stability, such as phosphorylation, ubiquitination and SUMOylation, which link to the ubiquitin-proteasome system [8]. A few E3 ubiquitin ligases have been shown to play roles in modulating ER alpha signaling. For example, BRCA1 and MDM2 could ubiquitinate ER alpha and trigger proteasomal degradation [6, 7, 9]. Interestingly, recent studies show that a few E3 ligases could modulates ER alpha signaling through stabilizing ER alpha protein, such as RNF31 and RNF8 [10–12].

Here, we identify the E3 ubiquitin ligase SMURF1 (SMAD Specific E3 Ubiquitin Protein Ligase 1) as one such kind of factor. SMURF1 was firstly identified as a negative regulator of SMADs (Mothers Against DDP homolog), which mediated SMADs ubiquitination and degradation [13]. In this study, we characterize a novel non-genomic regulatory mechanism that SMURF1 controls ER alpha ubiquitination and stability, which subsequently regulates estrogen-dependent gene expression and cancer cell proliferation.

## Results

### SMURF1 depletion inhibits ER alpha positive breast cancer cell proliferation in vitro and in vivo

**Fig. 1:**
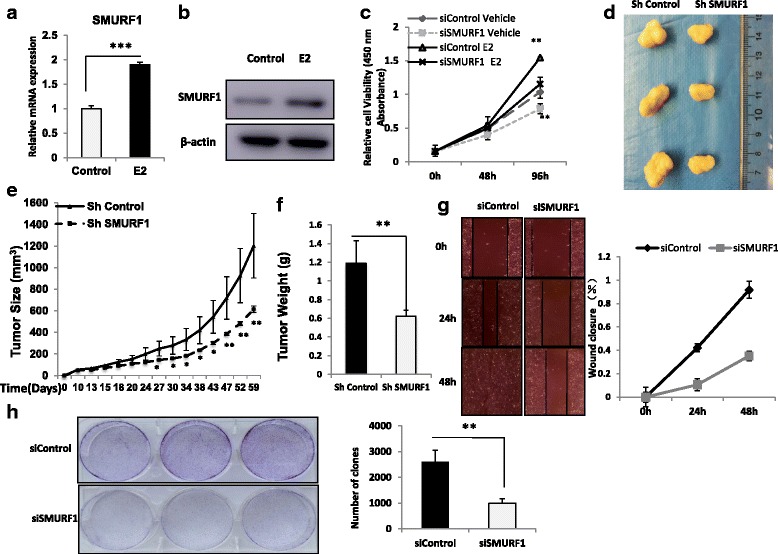
SMURF1 depletion inhibits ER alpha positive breast cancer cell proliferation in vitro and in vivo. **a** and **b** Estrogen stimulates the expression of endogenous SMURF1 in breast cancer cells. MCF-7cells are treated with 10 nM estradiol. After 24 h, SMURF1 mRNA and protein levels were determined by Western blot analysis. Actin was used as internal control. Experiments were done in triplicates. *P < 0.05; ** *P* < 0.01; ***P < 0.001 for SMURF1 mRNA level comparison. **c** SMURF1 depletion inhibits the cell proliferation in breast cancer cells. MCF-7 cells were transfected with 50 nM SMURF1 siRNA (mix of #1 and #2) or 50 nM control siRNA. After 24 h, the WST assay was used to determine the cellar metabolic activity at indicated time points after transfection. Cells are treated for indicated times with 10 nM E2 or vehicle. Experiments were done in triplicates. *P < 0.05; ** *P* < 0.01; ***P < 0.001 for cell growth comparison. **d**, **e** and **f** MCF-7 cells were stably transfected with lentivirus carrying scrambe shRNA or SMURF1 shRNA. Female NOD scid gamma (NSG) mice were estrogen-supplemented by implantation of slow-release 17β-estradiol pellets (0.72 mg/90-d release; Innovative Research of America) 1 day before MCF-7 tumor cell injection into the mammary fat pad (2 × 10^6^ MCF-7 cells suspended in 100ul Matrigel solution). MCF-7 tumor xenografts were measured every 3~5 days and the tumor volume were calculated by length × width^2^ /2. The mice were sacrificed at 2 month after transplant, and the tumors were weighted. The tumor growth curve, photograph and tumor weight were shown in Figure (**d**), (**e**) and (**f**) respectively. **g** Wound healing assay of MCF-7 transfected with the indicated siRNA. Quantification of wound closure at the indicated time points. Data are presented as ± SD. **, *P* < 0.01, ***, *P* < 0.001 (student’s t-test). **h** Clone formation assay of MCF-7 cells transfected with indicated siRNA. Quantification of clone formation is shown at the indicated time points. Data are presented as ± SD. **, *P* < 0.01, ***, *P* < 0.001 (student’s t-test)

By analyzing the public available microarray data in breast cancer, we observed SMURF1 mRNA level could be induce by estradiol treatment. In our experiments, we observed that 10 nM estradiol could increase SMURF1 in both mRNA and protein level (Fig. 1a and b). Published ChIP sequqnce data showed ER alpha could bind to the promoter region of SMURF1 gene in the chromatin [14] (Additional file 1: Figure S1A). ChIP assay showed that both ER alpha and H3K27-acetylated protein could bind to the indicated promoter region (Additional file 1: Figure S1B). In order to investigate the role of SMURF1 in breast cancer cells, SMURF1 was depleted in MCF-7 and T47D cells. SMURF1 depletion decreased cell proliferation in both vehicle and estradiol treated conditions (Fig. 1c and Additional file 1: Figure S2A). Next, we investigated the role of SMURF1 in tumor growth by xeno-graft mice models. Our data showed that SMURF1 depletion by lent-virus based shRNA decelerated breast tumor growth (Fig. 1d–f, Additional file 1: Figure S2B and C). Besides, depletion of SMURF1 significantly decreased cell migration capacity and clone formation capability in both MCF-7 and T47D cells (Fig. 1f, g, Additional file 1: Figure S3A and B).

### SMURF1 depletion decreases the expression of ER alpha target genes in breast cancer cells

**Fig. 2:**
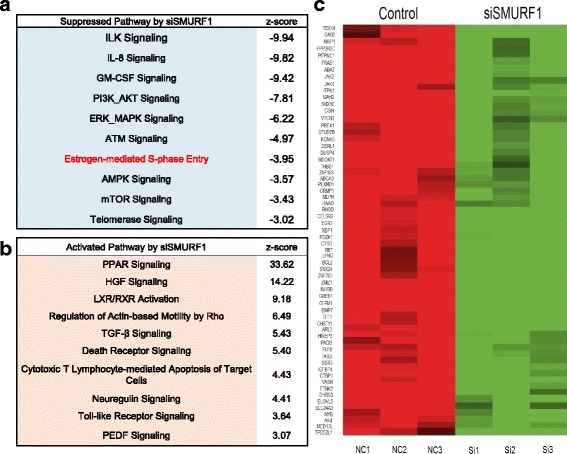
SMURF1 depletion decreases the expression of ER alpha target genes in breast cancer cells. **a** Top 10 signaling pathways significantly decreased by SMURF1 depletion in MCF7 cells. The pathway-enrichment analysis was used by the threshold P < 0.001 and fold change > 2 to derive regulated genes. SMURF1 was depleted by siRNA (mix of siSMURF1 #1 and siSMURF1 #2) or treated with siControl. After 48 h, the whole mRNA was extracted for RNA sequence analysis. The siControl and siSMURF1 were done in triplicates. **b** Top 10 signaling pathways, which were significantly activated by SMURF1 depletion in MCF7 cells. The pathway-enrichment analysis was used by the threshold P < 0.001 and fold change > 2 to derive regulated genes. The pathway-enrichment analysis was used by the threshold *P* < 0.001 and fold change > 2 to derive regulated genes. SMURF1 was depleted by siRNA (mix of siSMURF1 #1 and siSMURF1 #2) or treated with siControl. After 48 h, the whole mRNA was extracted for RNA sequence analysis. The siControl and siSMURF1 were done in triplicates. **c** The heat-map graph shows the ERα regulating genes, which is significantly changed by SMURF1 depletion in MCF-7 cells

To approach the function of SMURF1 in breast cancer cells in an unbiased way, we depleted SMURF1 in MCF-7 breast cancer cells for the whole genomic expression analysis. By comparison with siControl cells, SMURF1 depletion was associated with several changes in specific signaling pathways. Pathway analysis showed that SMURF1 depletion decreased ILK signaling (Integrin-linked kinase), AKT signaling, MAPK signaling, ER alpha signaling and so on (Fig. 2a). On the other hand, SMURF1 depletion also activated several signaling including PPAR signaling and TGF beta signaling (Fig. 2b). Since ER alpha signaling is pre-dominant in ER alpha positive breast cancer cells, we further analyzed ER alpha target genes expression change by SMURF1 depletion. It was shown that SMURF1 depletion significantly decreased ER alpha target gene expression, including GREB1, PS2 and PDZK1 (Fig. 2c and Additional file 1: Table S2).

### SMURF1 depletion or inhibition in breast cancer cells decreases ER alpha signaling activity

**Fig. 3:**
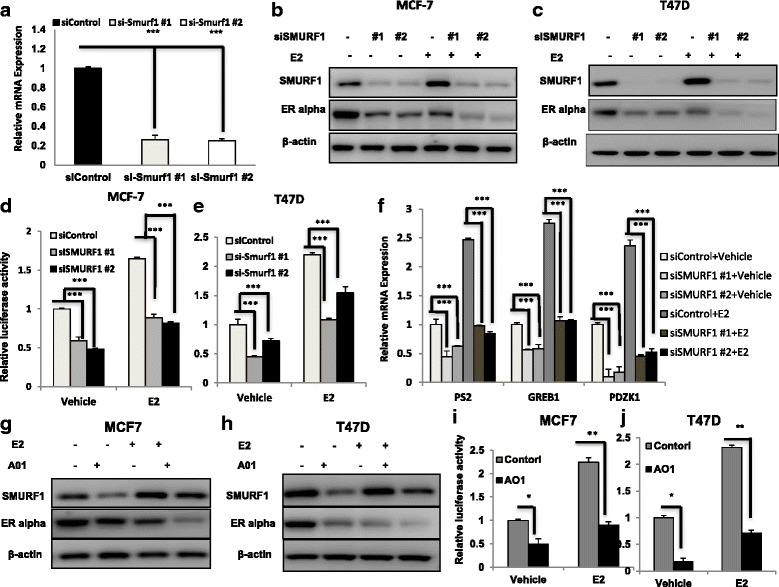
SMURF1 depletion or inhibition in breast cancer cells decreases ER alpha signaling activity. **a** SMURF1 depletion effect by two different siRNA oligos. MCF-7 cells are transfected with two independent SMURF1 siRNAs or siControl. After 48 h, SMURF1 mRNA levels are determined by QPCR. 36B4 was used as internal control. *P < 0.05; ** *P* < 0.01; ***P < 0.001 for SMURF1 mRNA level comparison. **b** and **c** SMURF1 depletion effect on ERα protein level by two different siRNA oligos. MCF-7 or T47D cells were transfected with siSMURF1 or siControl. After 48 h, cells were treated with either ethanol or 10 nM estradiol for 6 h. SMURF1 and ERα protein levels were determined by Western blot analysis. Actin was used as internal control. **d** and **e** SMURF1 depletion affects ERE-luciferase activity in MCF7 and T47D cells. MCF7 or T47D cells were transfected with siSMURF1 or siControl together with ERE luciferase reporter plasmid. Cells were treated with 10 nM estradiol or vehicle. Luciferase activity was measured 48 h after transfection. Shown are the results from three experiments. *P < 0.05; ** *P* < 0.01; ***P < 0.001 for luciferase activity comparison. **f** SMURF1 depletion decreases ERα target genes using two different siRNA oligos. MCF-7 cells were transfected with siSMURF1 or siControl. After 48 h, cells were treated with either ethanol or 10 nM estradiol for 6 h. Total RNA was prepared and the expression of the endogenous ERα target genes, PS2, GREB1, and PDZK1 were determined by qPCR. Shown are the results from three experiments. *P < 0.05; ** *P* < 0.01; ***P < 0.001 for target gene expression comparison. **g** and **h** SMURF1 inhibitor A01 decreases ER alpha protein in breast cancer cells. MCF-7 and T47D cells were both treated with 10 nM E2 or vehicle for 24 h and then continue with 10 uM A01 or vehicle 12 h,ER alpha and SMURF1 levels were determined by western blot analysis. β-actin was used as internal control. **i** and **j** SMURF1 inhibitor A1 decrease ERE-luciferase activity in MCF7 and T47D cells. MCF7 or T47D cells were transfected with ERE luciferase reporter plasmid. Cells were both treated with 10 nM E2 or vehicle for 24 h and then continue with 10 uM A01 or vehicle for 12 h. Luciferase activity was measured. Shown are the results from three experiments. *P < 0.05; ** *P* < 0.01; ***P < 0.001 for luciferase activity comparison

We further addressed the consequences of SMURF1 depletion in ER alpha signaling, which linked to ER alpha positive cancer proliferation. Two different individual siRNAs showed that SMURF1 depletion decreased ER alpha protein level in MCF-7 and T47D cells (Fig. 3a-c). To determine if SMURF1 depletion affect ER alpha transcriptional activity, we measured ER alpha reporter gene activity by SMURF1 depletion. Fig. 3d and e showed that SMURF1 depletion decreases ER alpha reporter gene activity in the presence and absence of estrogen in MCF-7 and T47D cells. Consistent with this, SMURF1 depletion also reduced the expression of endogenous ER alpha target genes such as PS2, GREB1 and PDZK1 in MCF-7 and T47D cells (Fig. 3f and Additional file 1: Figure S4A). In order to confirm the role of SMURF1 in ER alpha signaling, we utilized one selective SMURF1 inhibitor –A01 to treat breast cancer cell lines [15]. In consistent with SMURF1 depletion effect, A01 decreased ER alpha protein level in both MCF-7 and T47D cells (Fig. 3g and h). Besides, A01 treatment decreased ER alpha reporter gene activity (Fig. 3i and j). Real-time PCR experiments showed that A01 treatment also decreases ER alpha target gene expression such as GREB1, PS2 and PDZK1 in MCF-7 and T47D cells (Additional file 1: Figure S4B and C).

### SMURF1 associates with ER alpha and increases its stability

**Fig. 4:**
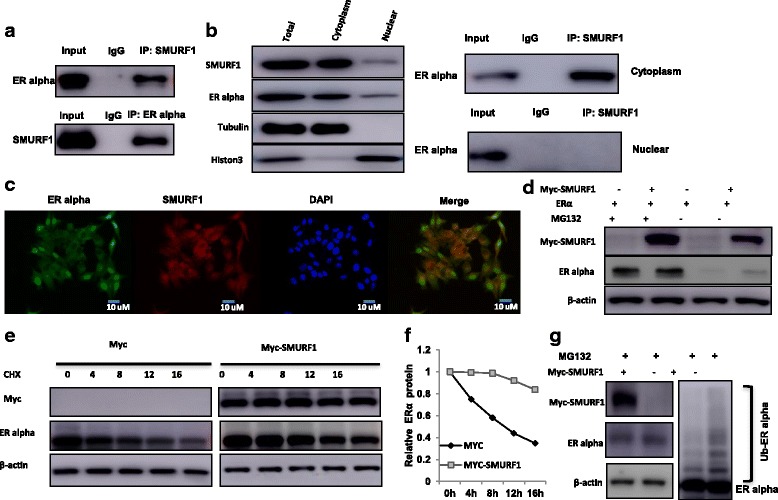
SMURF1 associates with ER alpha and increases its stability. **a** Co-IP assay reveals association between endogenous SMURF1 and ERα in MCF7 cells. MCF-7 cells were harvested with NP-40 lysis buffer. CO-IP was performed using antibody as indicated. **b** SMURF1 is mainly localized in the cytoplasm and associates with ER alpha in the cytosol. The subcellular protein fractionation kit (Thermo scientific, 78,840) was used for cytoplasm and nuclear separation. Tubulin and Histone-3 were used for cytoplasm and nuclear control. Based on the separation, IP was done by SMURF1 antibody in both the cytosol and nuclear lysis. ER alpha antibody was used to detect the interaction in both the cytosol and nuclear. **c** Intracellular localization analysis of SMURF1 and ER alpha by immunofluorescence assay. MCF7 cells were cultured in phenol red-free DMEM medium. Intracellular localization of SMURF1 (red) and ER alpha (green) were shown. Nuclei (blue) were stained with 4′,6-diamidino-2-phenylindole (DAPI). **d** In the presence of the proteasome inhibitor MG132, the stabilization effect of SMURF1 on ER alpha did not further increase ER alpha protein levels. HEK293 cells were transfected with 2 μg ERα plasmid and 0.5 μg Myc-tag or Myc-SMURF1 plasmids. After 24 h, cells were treated with 10 uM MG132/vehicle for 6 h. Cell lysates were prepared for Western blot analysis. The results are representative for three independent experiments. **e** and **f** SMURF1 increases ERα half-life in HEK293 cells. HEK293 cells were transfected with HA-ERα plasmid and Myc tag or Myc-SMURF plasmids. After 24 h, cells were treated with 100 μM cycloheximide/vehicle for indicated times. Cell lysates were prepared for Western blot analysis. The results are representative for three independent experiments. The ER alpha relative density was measured by Image J software. **g** SMURF1 prohibits ERα poly-ubiquitination. HEK293 cells were transfected with 2 μg ERα plasmid and 0.5 μg Myc-tag or Myc-SMURF1 plasmids. After 24 h, cells were treated with 10 uM MG132 or vehicle for 6 h. Cells were directly harvested and Western blot analysis using ERα antibody was used to detect ubiquitinated ERα forms. The predicted molecular weight of polyubiquitinated ERα is indicated

Further support for the functional cooperation of SMURF1 and ER alpha was obtained from co-immunoprecipation (co-IP) of the endogenous proteins from MCF-7 cells. Co-IP showed that SMURF1 could interact with ER alpha (Fig. 4a). However *E. Coli* based protein expression coupled with pull-down assay failed to detect the direct interaction between ER alpha and SMURF1 (Additional file 1: Figure S5). Nuclear and cytoplasmic separation based co-IP showed that SMURF1 as a cytoplasmic protein interacts with ER alpha in the cytoplasm (Fig. 4b). Immuno-staining result showed that ER alpha localized both in the cytosol and nuclear under E2-free conditions, while SMURF1 mainly localized in the cytosol (Fig. 4c). Since it is well known that ER alpha could regulate its own expression in MCF-7 cells, making it difficult to distinguish direct effect of SMURF1 on ER alpha protein or mRNA levels in the cell line [16]. Thus we performed the protein stability assay in HEK293 cells. Upon inhibition of protein synthesis by cycloheximide, SMURF1 overexpression significantly increased ER alpha protein stability (Fig. 4e, f and Additional file 1: Figure S6). In the presence of the proteasome inhibitor MG132, the stabilization effect of SMURF1 on ER alpha did not further increase ER alpha protein level (Fig. 4d). The ubiquitin WB assay showed that overexpressed SMURF1 could significantly decrease ER alpha poly-ubiquitination chains (Fig. 4g). Interestingly, TGFβ stimulation did not significantly change ER alpha protein level, which means the regulatory role of SMURF1 on ER alpha is not dependent on TGFβ signaling (Additional file 1: Figure S7A).

### SMURF1 interacts with ER alpha AF1 domain through its HECT domain and prohibits ER alpha K48 specific poly-ubiquitination

**Fig. 5:**
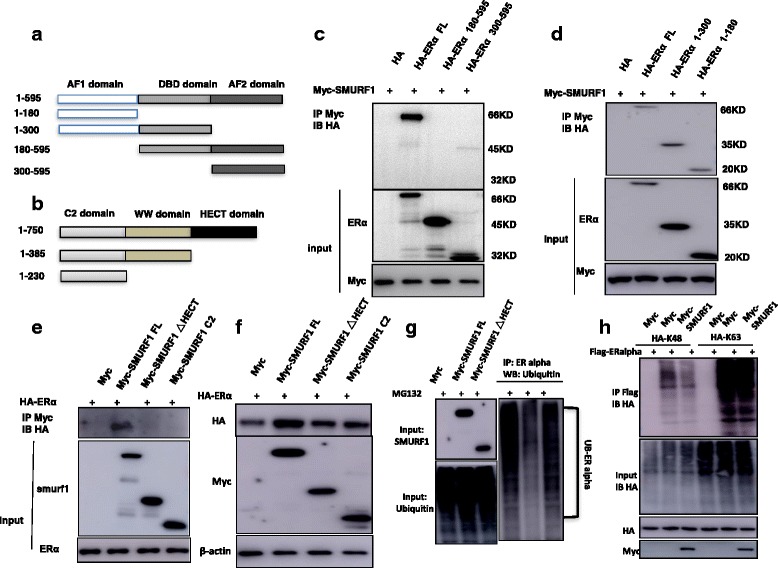
SMURF1 interacts with ER alpha AF1 domain throμgh its HECT domain and prohibits ER alpha K48 specific poly-ubiquitination. **a** ER alpha domain structure and deletion mutants used in the study (Full length, ΔAF1, ΔAF1 + ΔDBD, ΔAF2, ΔAF2 + ΔDBD). **b** SMURF1 domain structure and deletion mutants used in the study (Full length, ΔHECT, ΔHECT + ΔWW). **c** and **d** SMURF1 interacts with ER alpha throμgh its AF1 domain. HEK293 cells were transfected with 2 μg Myc-SMURF1 together with HA-ER alpha full length or mutants (ΔAF1, ΔAF1 + ΔDBD, ΔAF2 and ΔAF2 + ΔDBD). After 24 h, cells were harvested with NP-40 lysis buffer. CO-IP was performed using Myc antibody. The possible interacted ER alpha domains were detected by HA antibody. **e** HECT domain is required for SMURF1 interaction with ER alpha. HEK293 cells were transfected with 2 μg HA-ER alpha together with Myc-SMURF1 full length or mutants (ΔHECT, ΔHECT + ΔWW). After 24 h, cells were harvested with NP-40 lysis buffer. CO-IP was performed using HA antibody. The possible interacted SMURF domains were detected by Myc antibody. **f** The HECT domain is necessary for the SMURF1-mediated increase of ER alpha protein level. HEK293 cells were transfected with 2 μg HA-ER alpha together with Myc-SMURF1 full length or mutants (ΔHECT, ΔHECT + ΔWW). After 48 h, whole cell extracts were prepared and the level of ER alpha protein was assayed by western blot analysis. **g** The HECT domain is necessary for SMURF1 inhibition effect on ER alpha poly-ubiquitination. HEK293 cells were transfected with 2 μg Flag-ER alpha plasmid, 0.5 μg HA-Ub plasmid and 0.5 μg Myc-SMURF1/Myc-SMURF1_delta HECT/Myc-vector plasmids. The cell extracts were immunoprecipitated with ER alpha antibody. The poly-ubiquitinated ER alpha was detected by HA antibody. **h** SMURF1 decreases K48-linked poly-ubiquitination of ER alpha. HEK293 cells were transfected with 2 μg Flag-ER alpha plasmid, 0.5 μg HA-K48 Ubi/HA-K63 Ubi plasmid and 0.5 μg Myc-SMURF1 plasmids. The cell extracts were immunoprecipitated with HA antibody. The K48 specific poly-ubiquitinated ER alpha or K63 specific poly-ubiquitinated was detected via western blotting analysis

ER alpha has three functional domains: Activation Domain 1 (AF1), DNA binding domain (DBD) and Activation Domain 2 (AF2). SMURF1 has three functional domains: E6-AP Carboxyl Terminus domain (HECT), WW domain and C2 domain (Fig. 5a and b). We made these deletion constructs in order to delineate the interaction between ER alpha and SMURF1. The full length of ER alpha or ER alpha deletion constructs (ΔAF1 domain, ΔAF1 + ΔDBD domain, ΔAF2 domain, ΔAF2 + ΔDBD domain) was expressed together with SMURF-1 in HEK293 cells. Co-IP assay indicated that AF1 domain (1–180) was required for ER alpha to interact with SMURF1 (Fig. 5c and d). On the other hand, the full length of SMURF1 or deletion constructs (ΔHECT domain, ΔHECT + ΔWW domain) was expressed together with ER alpha in HEK293 cells. Co-IP assay showed that HECT domain of SMURF1 was necessary for its interaction with ER alpha (Fig. 5e). By overexpression SMURF1 full length or deletion constructs (ΔHECT domain, ΔHECT + ΔWW domain) together with ER alpha into HEK293 cells, we found that HECT domain was required for SMURF1 to exert its stabilization effect on ER alpha protein, while the HECT-dependent stabilization effect could be diminished by MG132 (Fig. 5f and Additional file 1: Figure S7B). Besides, deletion of HECT domain could not decrease the poly-ubiquitination of ER alpha compared with SMURF1 full length (Fig. 5g). As an ubiquitin ligase, SMURF1 possibly exerted its function via an ubiquitin-based manner. Thus we examined SMURF1 ubiquitination activity on ER alpha protein in two common ubiquitination manners (K48 and K63). Ubiquitin based immuno-precipitation assay showed that SMURF1 could significantly decrease K48 dependent poly-ubiquitination on ER alpha protein (Fig. 5h).

## Discussion

**Fig. 6:**
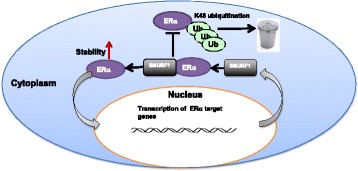
The hypothetical model for the positive feedback loop between SMURF1 and ER alpha signaling in breast cancer cells: ER alpha signaling induces the expression of SMURF1, which subsequently stabilizes ER alpha via its HECT domain possibly by inhibiting ER alpha K48-linked polyubiqutination process

Here we report that the HECT family ubiquitin ligase SMURF1 associates with and stabilizes ER alpha protein in the cytoplasm in breast cancer cells, which subsequently leads to increased estrogen signaling activity and cell proliferation. Interestingly, SMURF1 gene expression is also inducible by estrogen signaling, suggesting a forward feedback loop (Fig. 6). On this basis, SMURF1 inhibition, which breaks the positive feedback loop, could be a strategy to inhibit cell proliferation in ER alpha positive breast cancers.

ER alpha belongs to the nuclear receptor superfamily of transcription factors, and specifically to the ligand-dependent subfamily [17]. The basal and ligand-induced ER alpha protein levels are under tight control by ubiquitin-proteasome system [18]. Several ubiquitin ligases were shown to promote ER alpha protein degradation via facilitating ER alpha polyubiquitination, including CHIP and MDM2 [6, 9]. For example, CHIP protein was shown to associate with unliganded ER alpha and promotes its degradation. Interestingly, recently studies showed a group of E3 ligases exerted their stabilization effect on ER alpha protein through nonproteolytic ubiquitin manner [10]. For example, RNF8 was shown to interact with ER alpha in the nuclear and promote ER alpha mono-ubiquitination. Our previous studies also showed RNF31 could stabilize ER alpha via inducing ER alpha mono-ubiquitination [10, 11]. In our current study, we show that SMURF1 is involved in a positive feedback and possibly functions to maintain ER alpha stability. SMURF1 associates with the N terminal of ER alpha in the cytoplasm and prohibits ER alpha K48 poly-ubiquitination. There are two possible models to explain this stabilization effect. One is that SMURF1 exerts its function in an ubiquitin modification dependent manner. Another is that SMURF1 binds to ER alpha and prohibits its binding to other proteolytic E3 ubiquitin ligase. Based on our current assay, we do not found the known ubiquitination manner on ER alpha, which is significantly induced by SMURF1, including K63, K48 and Monoubiquitination (data not shown). However, our data indicate SMURF1 interacts with ER alpha AF1 domain, which account for a possible explanation for the stabilization effect. Previous studies showed that a few ER alpha co-activators could bind to AF1 domain and exert its stabilization effects, such as PIN1, MUC1 and ABL [19–21].

SMURF1 belongs to the HECT-type E3 ligases family proteins, which was firstly discovered as the antagonist of transforming growth factor beta (TGF beta) signaling [22]. SMURF1 interacts with several SMADs and promotes their poly-ubiqutination and degradation. Besides, SMURF1 is also shown to participate in Wnt signaling by promoting Prickle protein poly-ubiquitination and degradation [23]. However, other studies in SMURF1 also indicate that SMURF1 could also modulate certain protein in a nonproteolytic manner. For example, SMURF1 could stabilize MDM2, which subsequently promotes P53 degradation [24]. Besides, SMURF1 could also mediate K29-linked nonproteolytic polyubiquitination of axin in Wnt signaling [25]. In our study, we identify SMURF1 exerts its stabilization effect on ER alpha protein, which effect is dependent on HECT domain. Thus it is a bit contradictory that HECT domain is known to be a catalytic domain for E3 ubiquitin ligase. It remains to be determined that why SMURF1 exerts different protein stability consequences on its target proteins. Our study also indicates SMURF1 is one of ER alpha signaling target genes, which could be transcriptional increased by estradiol. However, previous study showed that ER alpha could promote SMURF1 protein degradation in MCF-7 and HEK293 cells [26]. This might indicate ER alpha could regulate SMURF1 mRNA and protein level in opposite directions.

Although ER alpha has been well documented to have a critical in cancer progression in breast cancer, SMURF1 emerges to be a new component of ER alpha signaling in breast cancer. Beside, previous studies showed SMURF1 was recognized as an oncogene, since it was found to suppress several tumor suppressor pathways, such as TGF beta signaling and P53 signaling [24, 27]. Several clinical studies also showed that SMURF1 was amplified in the chromatin in pancreatic and esophageal carcinomas [28, 29]. Here, we identify the oncogenic role of SMURF1 in supporting estrogen signaling and breast cancer progression. Thus targeting SMURF1 could be a promising strategy or drug target for several kinds of human carcinomas, including ER alpha positive breast cancer.

## Methods

### Cell culture

MCF-7 and HEK293 cells were cultured in DMEM (Invitrogen, Carlsbad, CA) supplemented with 10% fetal bovine serum (FBS) and 1% penicillin/streptomycin (Invitrogen) at 37 °C in a humidified atmosphere of 5% CO2 in air. T47D cells were cultured in RPMI 1640 (Invitrogen) supplemented with 10% FBS (GIBCO) and 1% penicillin/streptomycin.

### Plasmids

The pRK-Myc-SMURF1 plasmid was acquired from Addgene (Plasmid #13676). The SMURF delta-HECT construct and delta-WW + HECT construct were sub-cloned from the original plasmid. The PcDNA3-HA-ER alpha plasmid was acquired from Addgene (Plasmid #49498). The ER alpha delta-AF1 construct, delta-AF1 + DBD construct, delta-AF2 construct and delta-AF2 + DBD construct were sub-cloned from ER alpha original plasmid. The HA-K48 and HA-K63 Ubi plasmids were gifted from Dr. Bo Yang and Jie Wang [30]. The flag-ER alpha plasmid was described from previous paper [10]. The ERE-TK-luc reporter and the pRL-TK control were described in previous study [31].

### siRNA and plasmids transfection

Cells were transfected with 50 nM siRNA. SMURF1 siRNAs sequences were shown here: SMURF1 siRNA #1: CCAGUAUUCUACGGACAAUdTdT; siRNA #2: CAUGAAAUGCUGAAUCCUUdTdT. Control siRNA sequences were shown: UUCUCCGAACGUGUCACGUTT. INTERFERin transfection reagent (Polyplus Transfection, 409–10) was used according to the manufacturer’s protocol. Plasmids were transfected by Lipofectamin 2000 (1,662,298, Invitrogen).

### RNA extraction and qPCR analysis

RNeasy kits were used to extract total RNA (Qiagen). qPCR was performed as previously described. 36B4 was used as internal control. Primer sequences for qPCR are provided in Additional file 1: Table S1.

### Quantification of cell viability

MCF-7 and T47D cells were transfected with siSMURF1 or siControl in 24-well plates. After 24 h, the cells were seeded into 96-well plates. Estrogen and vehicle were added in each group. Cell numbers were determined using the WST-1 cell proliferation reagent as previously described [32].

### Xenograft tumor model

MCF-7 cells were infected with shControl virus or shSMURF1 virus (SC108080 and SC41673V). After 48 h of infection, cells were treated with 2μg/ml puromycin for 3 days. Female nonobese diabetic-SCID mice were implanted with slow-relase 17 beta-estradiol pellets (0.72 mg/90-day, Innovative Research of America). After 1 day, each mouse was injected with 1X10^6^ MCF-7 cells together with matrigel solution into the mammary fat pad. The tumor sizes were measured each 3–5 days. After 2 mouths, the mice were sacrificed and the tumors were fixed with Formalin for measuring weight and photograph. The experiments were performed under the protocols approved by ethnic committee of Xinxiang Medical University.

### Wound healing assay

MCF-7 and T47D cells were seeded and transfected with 50 nM SMURF1 siRNA or control siRNA. 24 h after transfection, cells were seeded into 6-well plates with 1% FBS with 100% confluence. One yellow pipette tip was used to make a straight scratch. The wound distance was measured at indicated time points and normalized with starting time point. Percentage wound recovery was expressed as: [1-(Width of the wound at a given time/width of the wound at *t* = 0)] × 100%.

### Clone formation assay

MCF-7 and T47D cells were plated in six-well plates overnight and treated with 50 nM SMURF1 siRNA or 50 nM siControl. After 24 h, the cells were washed with PBS, trypsinized and plated at low density (5000 cell/well in six-well plate). The cells were cultivated for 7 days and the medium was refreshed every two days. The colonies were stained with crystal violet. The number of the clones in a given area was counted for each condition.

### Western blotting

Cells were lysed with RIPA lysis buffer. Anti- ER alpha mouse (1D5, SC56833) was from Santa Cruz Biotechnology. Anti-ER alpha rabbit (D8H8, #8644) was from Cell Signaling Technology. Anti-SMURF1 (AB117552), anti-Myc mouse (AB32), anti-Myc rabbit (AB9106) and anti-FLAG (M2, ab48763) were acquired from Abcam. Anti-HA (MMS-101R) was acquired from COVANCE. Anti-actin (8H10D10) was acquired from Cell Signaling Technology.

### Immunoprecipitation

Immunoprecipitation was performed as previous described [33]. 100μg cell lysls were pre-cleared with ribbit IgG for 2 h and subsequently incubated with ER alpha antibody (D8H8, #8644) over night, while rabbit IgG was used as the negative control. The bounded protein was analyzed by Anti-SMURF1 antibody (AB117552). For the overexpression experiment, HEK293 cells were transfected with 5μg Myc-SMURF1 and ERɑ plasmid in 10 cm dish. Cell lysates were pre-cleared with IgG and subsequently incubate with Myc (AB9106) antibody or ERɑ (D8H8, #8644) antibody, while rabbit IgG was used as the negative control. The bound proteins were analyzed by western blotting.

### Chromatin immuno-precipitation (ChIP) assay

ChIP assay was performed in our previous study. MCF-7 cells were fixed for crosslinking for 30 min. After that, the cells was mixed with 0.1375 M glycine, washed with cold PBS/1 mM PMSF and scratched into PBS/1 mM PMSF for centrifuge. Then cells were treated by SDS lysis buffer and sonicated for 10 min (30 s on/off). Then the ChIP assay kit (Millipore, 17–295) was used for following steps. The following antibodies were used in the ChIP experiments: anti-H3K27AC (Ab4729) and anti-ERɑ rabbit (D8H8, #8644). The primer sequences for ChIP assay were shown: Forward- CAACCTCCAGCCATTCTCACT; Reverse- TGTCTCCATATGCTGGTGGTG.

### Immunostaining

MCF-7 cells cultured on sterile glass cover slips were fixed with 4% formaldehyde for 10 min. Then the cells were incubated in permeabilization buffer (0.3% Triton X100, in PBS) for 10 min. Ten percent donkey serum was added to suppress nonspecific antibody binding at room temperature for 30 min and primary antibodies were incubated overnight at 4 degree. Fluorochrome-conjugated secondary antibodies were added after wash in a dark chamber at room temperature. The slides were washed with PBS and mounted using mounting solution containing DAPI. Finally, slides were visualized with a NIKON80i fluorescent microscope. The antibodies were used as follows: anti-ER alpha (SC-130072, santa cruz); anti-SMURF1 (AB57573,Abcam).

### Protein stability assays

HEK293 cells (10^5^) were seeded into 24 well plates and transfected with 0.5μg ERɑ plasmid together with 0.5μg Myc-SMURF1 or empty vector. After 48 h, cells were treated with 100uM cycloheximide (C7698, Sigma) for indicated time points. Samples were subject to western blot for ERɑ degradation.

### Pull down assay

ERα fragments corresponding to amino acid (aa) 1–300 and aa 294–595 were individually expressed as his-fusion proteins using the His-CtsR system. His-tagged proteins were purified with nickel-nitrilotriacetic acide (Nii-NTA) resin. GST-fusion SMURF1 proteins were purified using glutathione-Sepharose beads (17–0756-01, GE Healthcare, Uppsala, Sweden) according to the Manufacturer’s protocol. The mixture was incubated at 4 °C with rotation for 30 min; the resin was washed twice with PBS containing 30 mM imidazole, followed by washing twice with PBS containing 0.01% Triton X-100. Bound proteins were eluted with elution buffer (50 mM sodium phosphate, 300 mM NaCl, 250 mM imidazole [pH 7.4]) and subjected to SDS-PAGE analysis.

### Analysis of protein ubiquitination

HEK293 cells were transfected with 2 μg ERɑ plasmid together with 2 μg Myc-SMURF1 or empty vector. After 48 h, cells were treated with 10uM MG132 (474,787, Sigma) or ethanol for 6 h. Cells were directly harvested. The poly-ubiquitination of ERɑ was detected by western blotting analysis.

### Poly-ubiquitination detection assay

To directly detect the enriched K48-ubiquitinated or K63-ubiqutinated ERɑ from the cell extracts, HEK293 cells were transfected with 0.5 μg K48 Ubi or 4 μg K63 Ubi plasmid, 2 μg ERɑ together with 0.5 μg Flag-SMURF1 or Flag-vector. After 48 h, total protein was extracted and pre-cleared by 20ul protein A (santa cruz, SC-2001) for 2 h. The supernatant was corrected and immunoprecipitated by HA antibody. Western blot with rabbit anti- ERɑ antibody was performed to detect K48 or K63 poly-ubiquitinated ERɑ.

### Luciferase assay

The luciferase activity was done using the Dual-Luciferase Reporter kit (Promega, Germany). The ERE luciferase reporter was transfect together with renila plasmid into the cells. The luciferase activity was measured after 24 h.

### RNA sequence analysis

The global gene expression analysis was based on RNA sequencing platform from BGI (Beijing Genomic Institute). The RNA sequence data are deposited in the Gene Expression Omnibus (GEO) database (Assessing number: GSE102653). Analysis was performed for differentially expressed genes (*P* < 0.01 and fold change >2) by Ingenuity Pathway Analysis (IPA).

### Statistics

Student’s t-test and Pearson correlation coefficient were used for comparisons. A *P*-value of <0.05 was considered to be significant.

## Conclusion

In conclusion, our study reveals a novel positive feedback between SMURF1 and ER alpha signaling in supporting breast cancer growth. Targeting SMURF1 could be one promising strategy for ER alpha positive breast cancer treatment.

## Additional file

Additional file 1: **Figure S1A.** Public available ChIP sequence data indicates that ER alpha could bind to SMURF1 promoter region at the first intron. **Figure S1B.** ChIP assay shows that ER alpha and H3K27AC are recruited to SMURF1 promoter region. **Figure S2A.** SMURF1 depletion inhibits the cell proliferation in breast cancer cells in T47D cells. **Figure S2B.** MCF-7 cells were stably transfected with lentivirus carrying scrambe shRNA (*N* = 4) or SMURF1 shRNA (*N* = 4). The mice were sacrificed at two month after transplant, and the tumors were weighted. The tumor growth curve and photograph were shown respectively. **Figure S3A.** Wound healing assay of T47D transfected with the indicated siRNA. **Figure S3B.** Clone formation assay of T47D cells transfected with indicated siRNA. **Figure S4A.** SMURF1 depletion decreases ERα target genes using two different siRNA oligos in T47D cells. **Figure S4B** and **C** SMURF1 inhibition decreases ERα target genes expression in MCF-7 and T47D cells. **Figure S5.** Pulldown assay shows that SMURF1 fails to directly interact with N-terminal or C-terminal of ER alpha. **Figure S6.** Three independent repeats of SMURF1 effect on ERα half-life in HEK293 cells. **Figure S7A.** TGFβ does not change ER alpha protein level in MCF-7 cells. MCF-7 cells were transfected with siSMURF1 or siControl. **Figure S7B.** HECT domain is required for the stabilization effect on ER alpha protein. **Table S1.** Primer sequences used in this study. **Table S2.** ER alpha target genes list by SMURF1 depleiton in MCF-7 cells. (PPTX 1594 kb)
