# Supplementary material for: SMURF1 facilitates estrogen receptor ɑ signaling in breast cancer cells
Source: J Exp Clin Cancer Res. 2018 Feb 12;37:24. doi: 10.1186/s13046-018-0672-z (PMC5808446; doi:10.1186/s13046-018-0672-z)
Supplement: Supplementary file 1 — Figure S1A. Public available ChIP sequence data indicates that ER alpha could bind to SMURF1 promoter region at the first intron. Figure S1B. ChIP assay shows that ER alpha and H3K27AC are recruited to SMURF1 promoter region. Figure S2A. SMURF1 depletion inhibits the cell proliferation in breast cancer cells in T47D cells. Figure S2B. MCF-7 cells were stably transfected with lentivirus carrying scrambe shRNA (N = 4) or SMURF1 shRNA (N = 4). The mice were sacrificed at two month after transplant, and the tumors were weighted. The tumor growth curve and photograph were shown respectively. Figure S3A. Wound healing assay of T47D transfected with the indicated siRNA. Figure S3B. Clone formation assay of T47D cells transfected with indicated siRNA. Figure S4A. SMURF1 depletion decreases ERα target genes using two different siRNA oligos in T47D cells. Figure S4B and C SMURF1 inhibition decreases ERα target genes expression in MCF-7 and T47D cells. Figure S5. Pulldown assay shows that SMURF1 fails to directly interact with N-terminal or C-terminal of ER alpha. Figure S6. Three independent repeats of SMURF1 effect on ERα half-life in HEK293 cells. Figure S7A. TGFβ does not change ER alpha protein level in MCF-7 cells. MCF-7 cells were transfected with siSMURF1 or siControl. Figure S7B. HECT domain is required for the stabilization effect on ER alpha protein. Table S1. Primer sequences used in this study. Table S2. ER alpha target genes list by SMURF1 depleiton in MCF-7 cells. (PPTX 1594 kb) [file 13046_2018_672_MOESM1_ESM.pptx]

## Slide 1
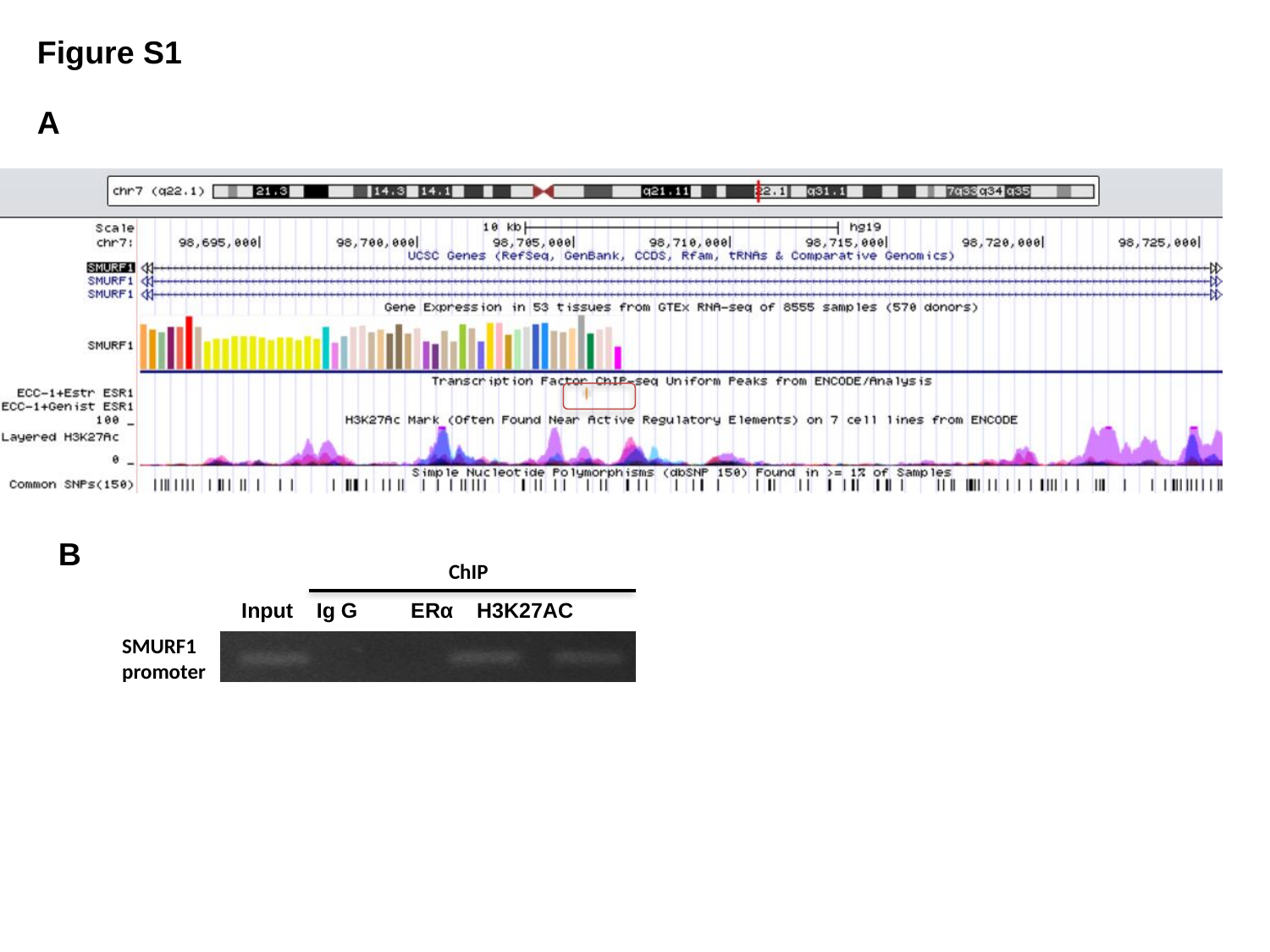

Figure S1
A
B
ChIP
Input Ig G ERα H3K27AC
SMURF1 promoter

## Slide 2
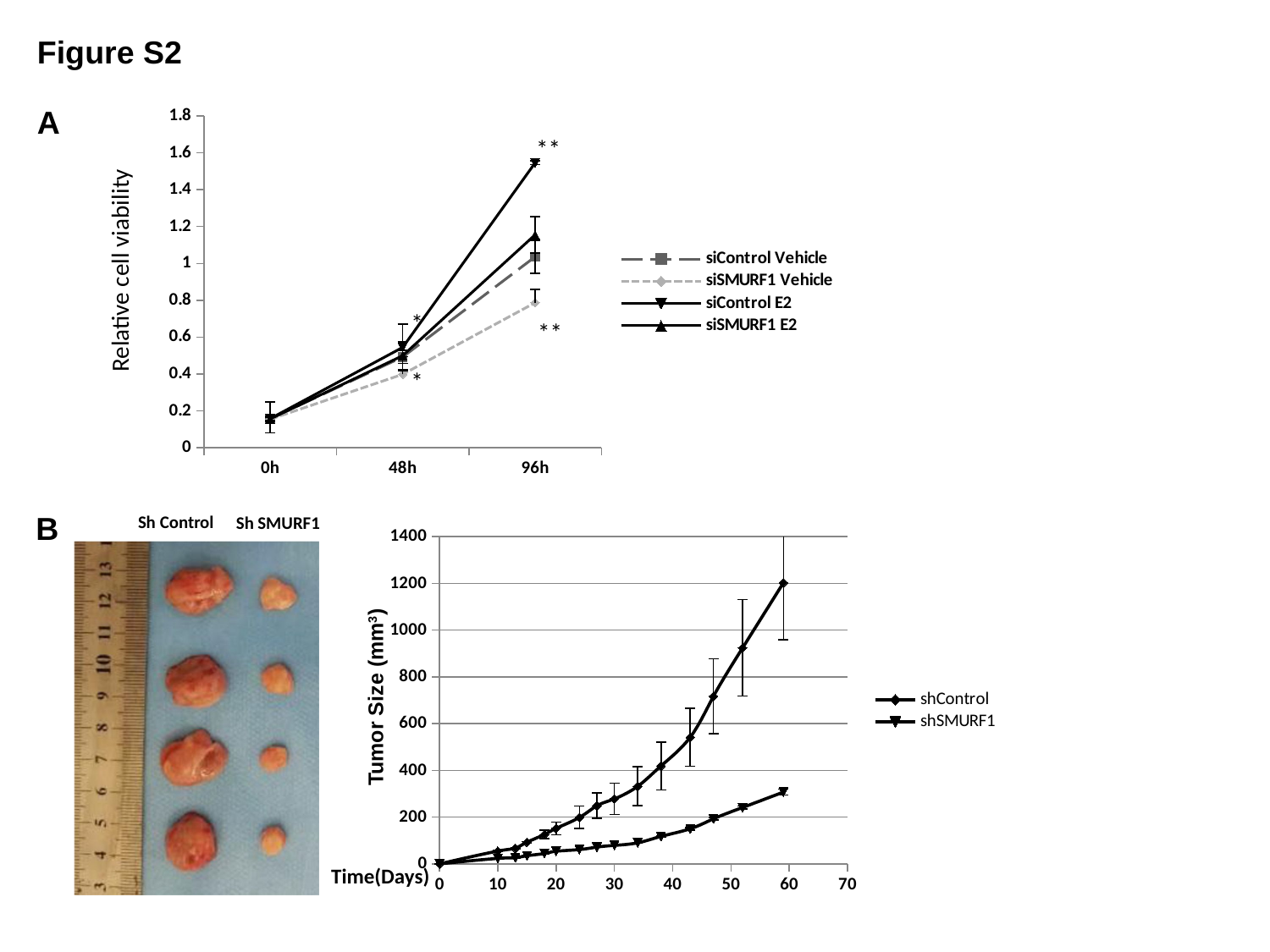

Figure S2
A
### Chart
| Category | siControl Vehicle | siSMURF1 Vehicle | siControl E2 | siSMURF1 E2 |
|---|---|---|---|---|
| 0h | 0.15680000000000002 | 0.15470000000000003 | 0.15780000000000002 | 0.15610000000000002 |
| 48h | 0.49250000000000005 | 0.3985000000000011 | 0.5446000000000001 | 0.49950000000000006 |
| 96h | 1.036 | 0.7868 | 1.5453999999999977 | 1.15525 |**
Relative cell viability
*
**
*
B
Sh Control
Sh SMURF1
### Chart
| Category | shControl | shSMURF1 |
|---|---|---|
Tumor Size (mm3)
Time(Days)

## Slide 3
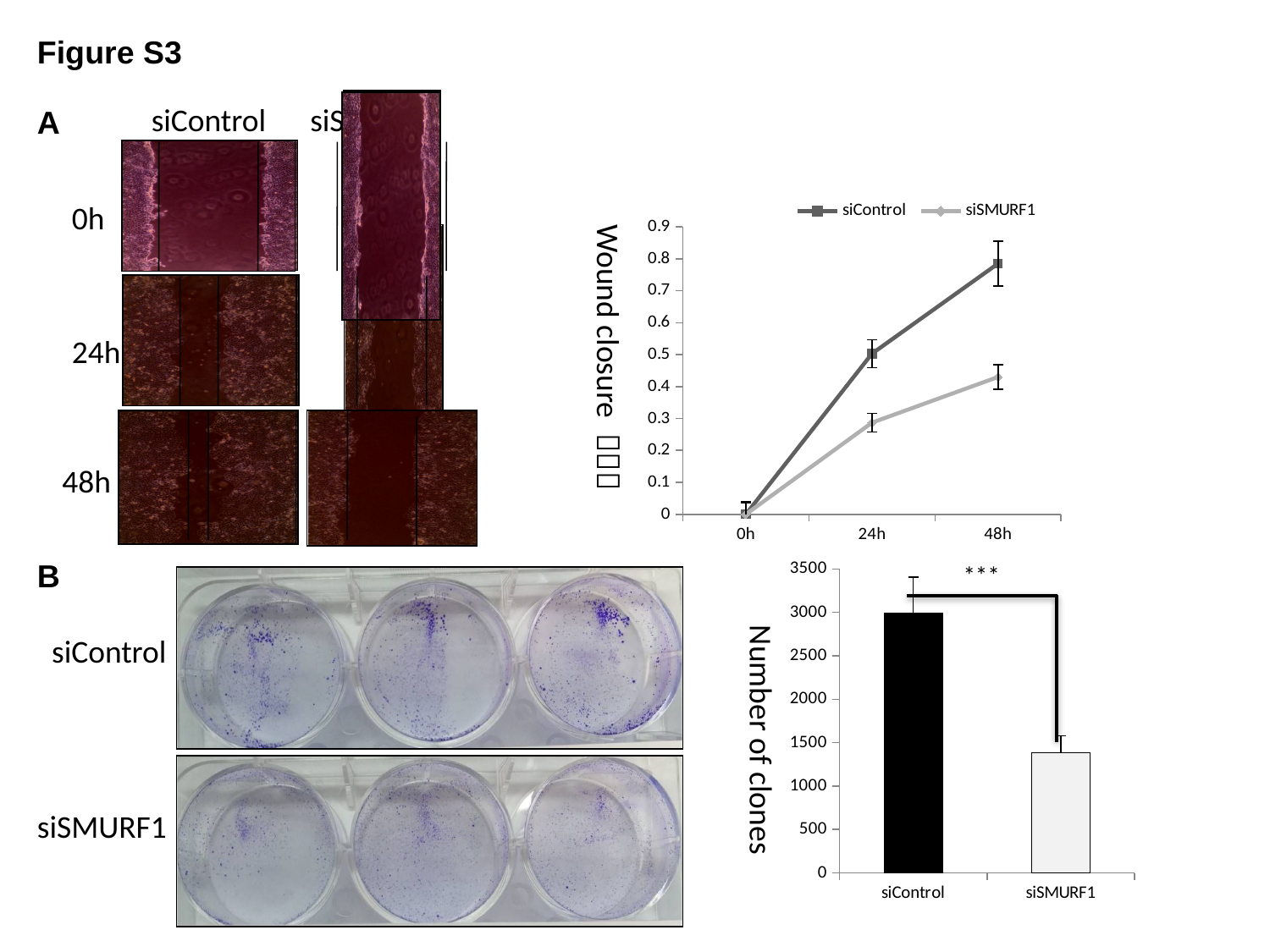

Figure S3
siSMURF1
siControl
0h
24h
48h
A
### Chart
| Category | siControl | siSMURF1 |
|---|---|---|
| 0h | 0.0 | 0.0 |
| 24h | 0.503 | 0.28700000000000003 |
| 48h | 0.785 | 0.43000000000000005 |Wound closure （％）
### Chart
| Category | T47D |
|---|---|
| siControl | 3000.0 |
| siSMURF1 | 1380.0 |B
***
siControl
siSMURF1
Number of clones

## Slide 4
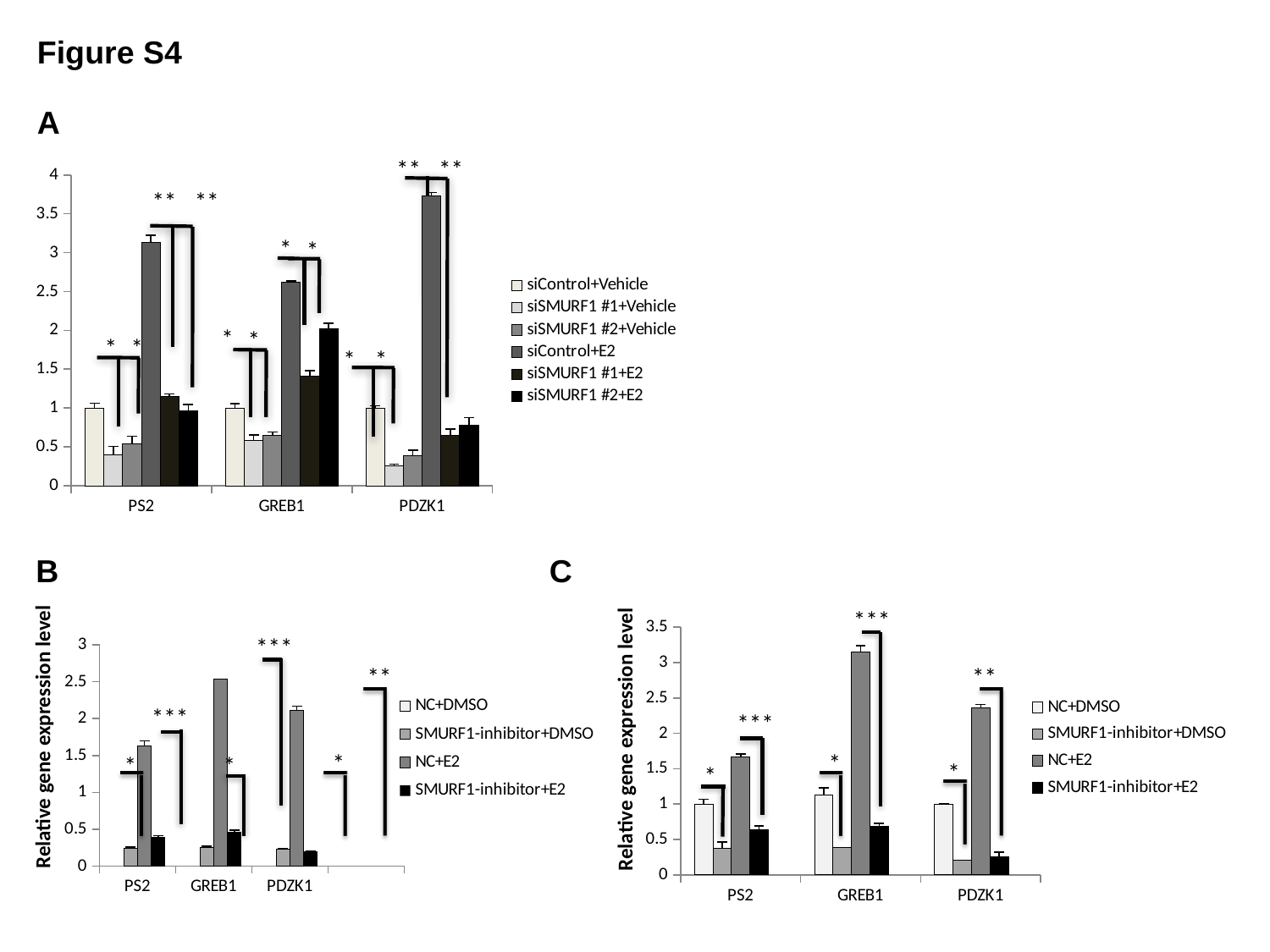

Figure S4
A
**
**
### Chart
| Category | siControl+Vehicle | siSMURF1 #1+Vehicle | siSMURF1 #2+Vehicle | siControl+E2 | siSMURF1 #1+E2 | siSMURF1 #2+E2 |
|---|---|---|---|---|---|---|
| PS2 | 1.0 | 0.39825420000000006 | 0.5346712000000001 | 3.1328149999999977 | 1.146528 | 0.9671685 |
| GREB1 | 1.0 | 0.5852105999999988 | 0.6457561 | 2.620943 | 1.414452 | 2.0222519999999977 |
| PDZK1 | 1.0 | 0.2508979 | 0.3910005000000001 | 3.727934000000003 | 0.6506193000000001 | 0.7786891000000001 |**
**
*
*
*
*
*
*
*
*
B
C
Relative gene expression level
Relative gene expression level
***
### Chart
| Category | NC+DMSO | SMURF1-inhibitor+DMSO | NC+E2 | SMURF1-inhibitor+E2 | |
|---|---|---|---|---|---|
| PS2 | 0.9999163352896508 | 0.3747019073424551 | 1.67079772019443 | 0.6427181461668902 | None |
| GREB1 | 1.132481 | 0.37989100000000003 | 3.150523 | 0.687000000000001 | None |
| PDZK1 | 1.0002375999470021 | 0.20484755393289203 | 2.3585951251450767 | 0.25272463692998304 | None |***
### Chart
| Category | NC+DMSO | SMURF1-inhibitor+DMSO | NC+E2 | SMURF1-inhibitor+E2 |
|---|---|---|---|---|
| PS2 | 1.0000001472229878 | 0.246173308503833 | 1.62954855717113 | 0.39998539527772514 |
| GREB1 | 1.000000000163598 | 0.25768814521499406 | 2.5312011845233777 | 0.46284016172143905 |
| PDZK1 | 0.999999898531957 | 0.225123417765356 | 2.110637432118973 | 0.19504949493136406 |**
**
***
***
*
*
*
*
*
*

## Slide 5
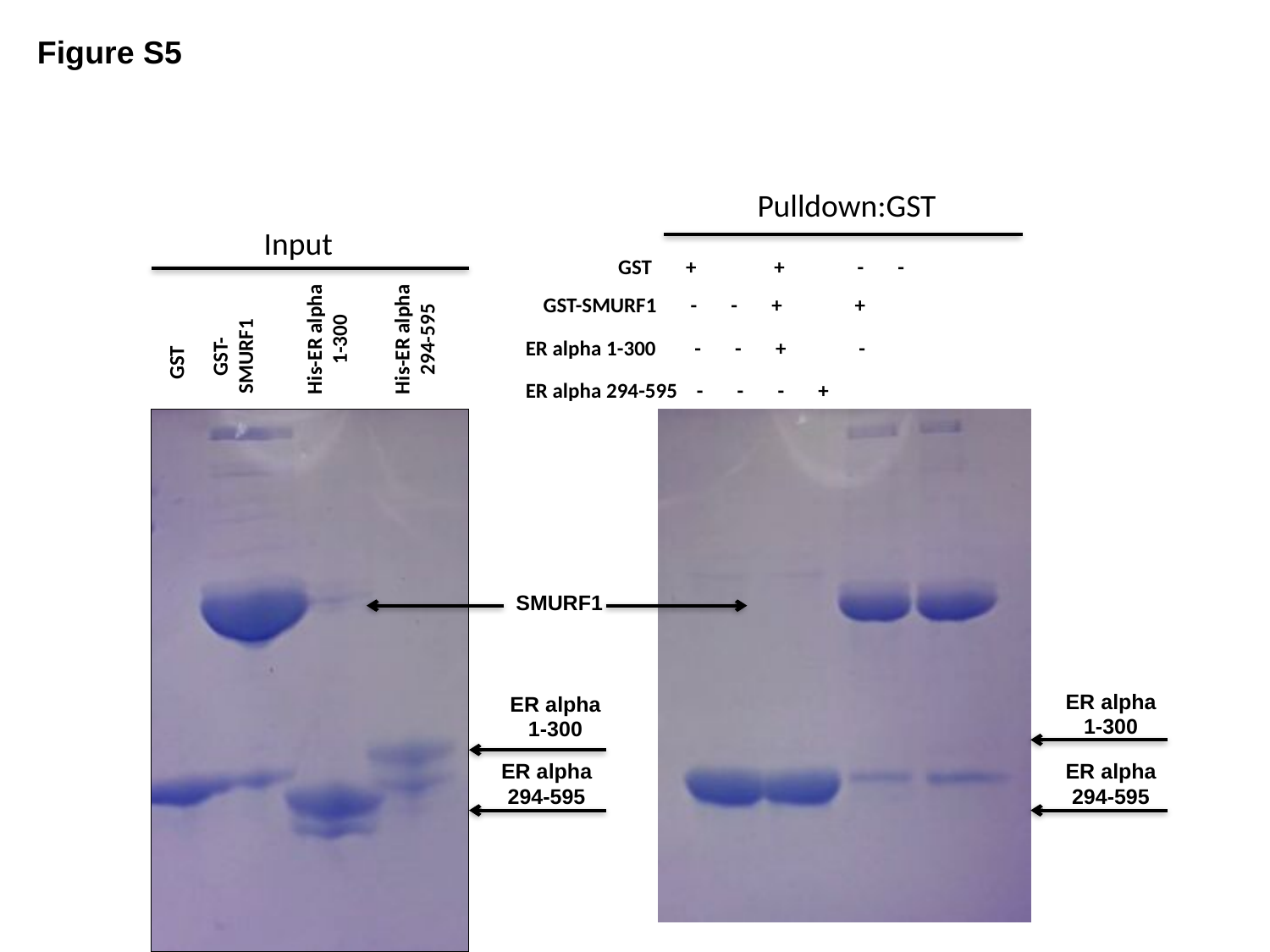

Figure S5
Pulldown:GST
Input
GST + + - -
GST-SMURF1 - - + +
His-ER alpha
294-595
His-ER alpha
1-300
GST
GST-SMURF1
ER alpha 1-300 - - + -
ER alpha 294-595 - - - +
SMURF1
ER alpha 1-300
ER alpha 1-300
ER alpha 294-595
ER alpha 294-595

## Slide 6
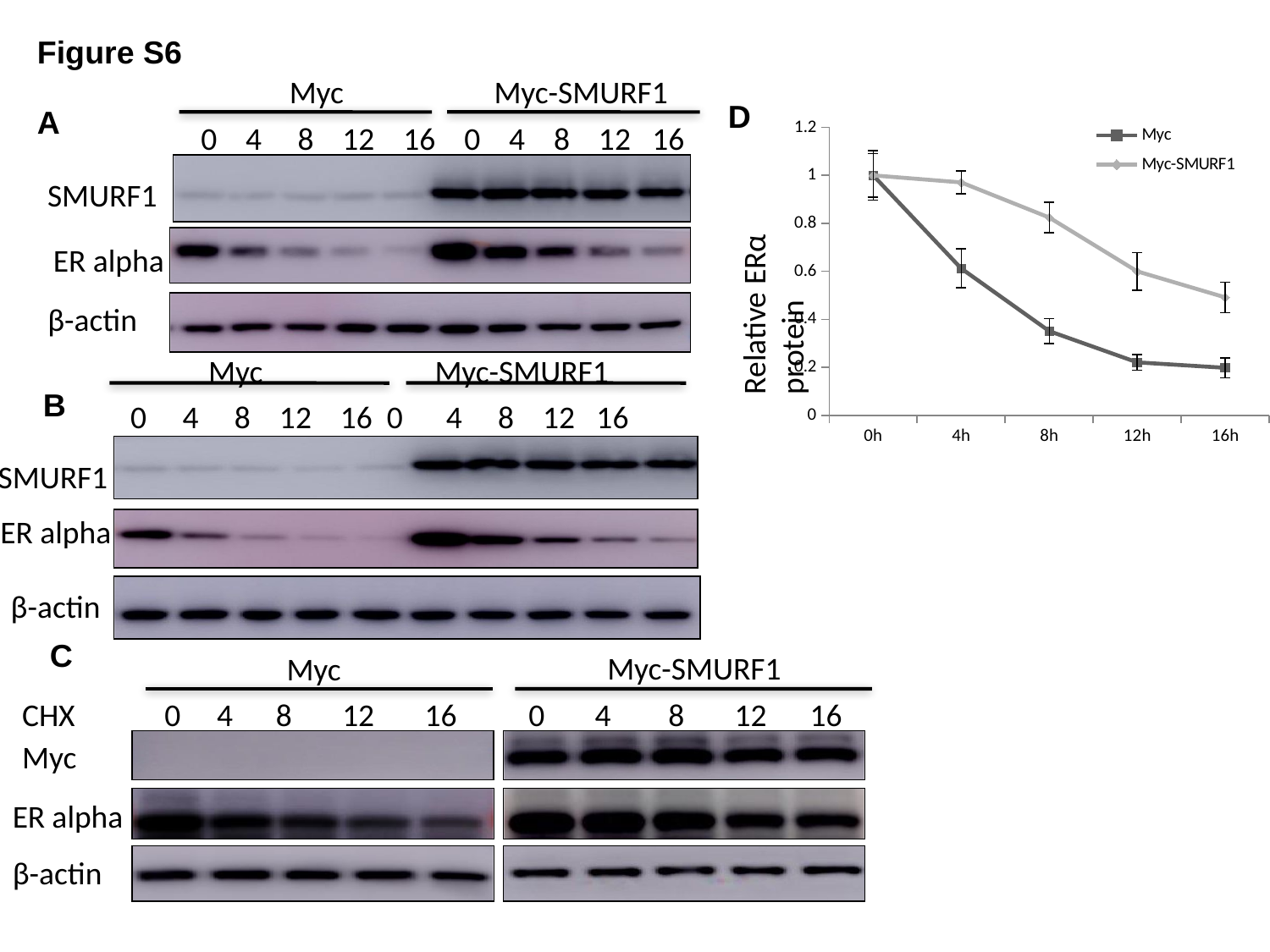

Figure S6
Myc
Myc-SMURF1
 0 4 8 12 16 0 4 8 12 16
SMURF1
ER alpha
β-actin
D
A
### Chart
| Category | Myc | Myc-SMURF1 |
|---|---|---|
| 0h | 1.0 | 1.0 |
| 4h | 0.6120929296591341 | 0.970607731800413 |
| 8h | 0.35076787374052604 | 0.8242295589305831 |
| 12h | 0.22052478015632804 | 0.6000344404545371 |
| 16h | 0.19797722822474198 | 0.49139743939233604 |Relative ERα protein
Myc
Myc-SMURF1
 0 4 8 12 16 0 4 8 12 16
SMURF1
β-actin
B
ER alpha
C
Myc-SMURF1
Myc
 0 4 8 12 16 0 4 8 12 16
CHX
Myc
ER alpha
β-actin

## Slide 7
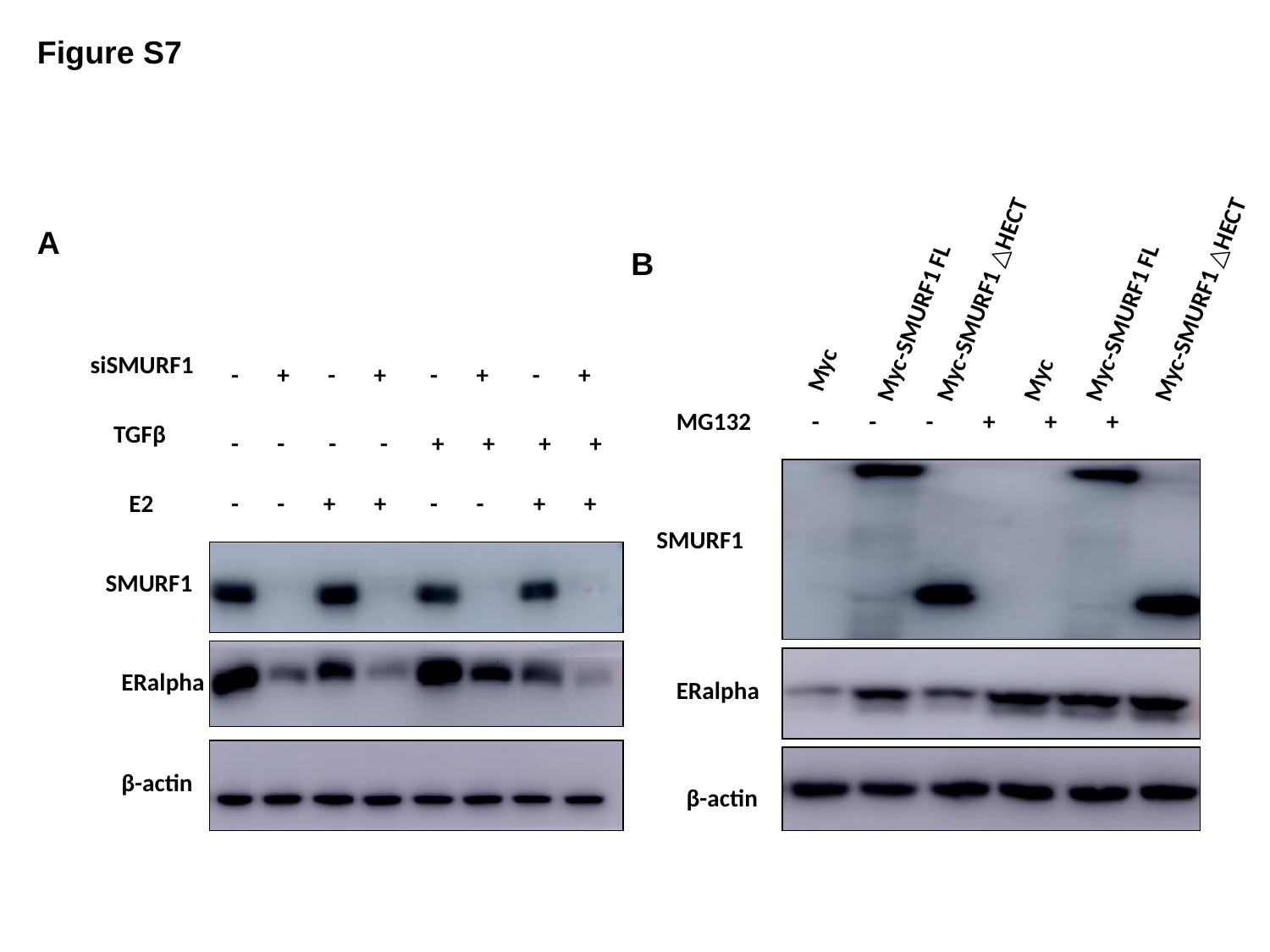

Figure S7
Myc
Myc-SMURF1 FL
Myc-SMURF1 FL
Myc-SMURF1 △HECT
Myc
Myc-SMURF1 △HECT
MG132
 - - - + + +
SMURF1
ERalpha
β-actin
siSMURF1
 - + - + - + - +
TGFβ
 - - - - + + + +
E2
 - - + + - - + +
SMURF1
ERalpha
β-actin
A
B

## Slide 8
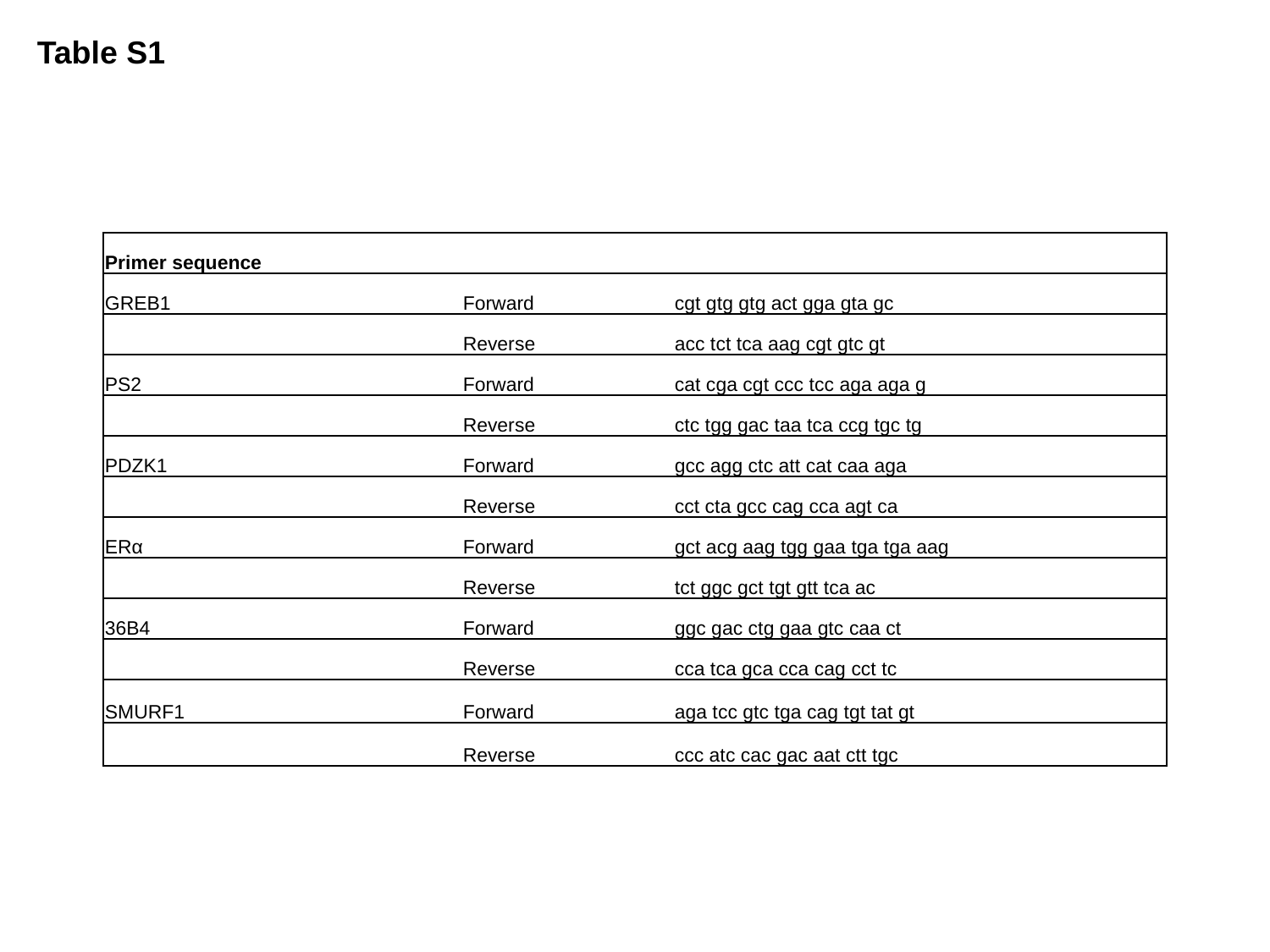

Table S1
| Primer sequence | | |
| --- | --- | --- |
| GREB1 | Forward | cgt gtg gtg act gga gta gc |
| | Reverse | acc tct tca aag cgt gtc gt |
| PS2 | Forward | cat cga cgt ccc tcc aga aga g |
| | Reverse | ctc tgg gac taa tca ccg tgc tg |
| PDZK1 | Forward | gcc agg ctc att cat caa aga |
| | Reverse | cct cta gcc cag cca agt ca |
| ERα | Forward | gct acg aag tgg gaa tga tga aag |
| | Reverse | tct ggc gct tgt gtt tca ac |
| 36B4 | Forward | ggc gac ctg gaa gtc caa ct |
| | Reverse | cca tca gca cca cag cct tc |
| SMURF1 | Forward | aga tcc gtc tga cag tgt tat gt |
| | Reverse | ccc atc cac gac aat ctt tgc |

## Slide 9
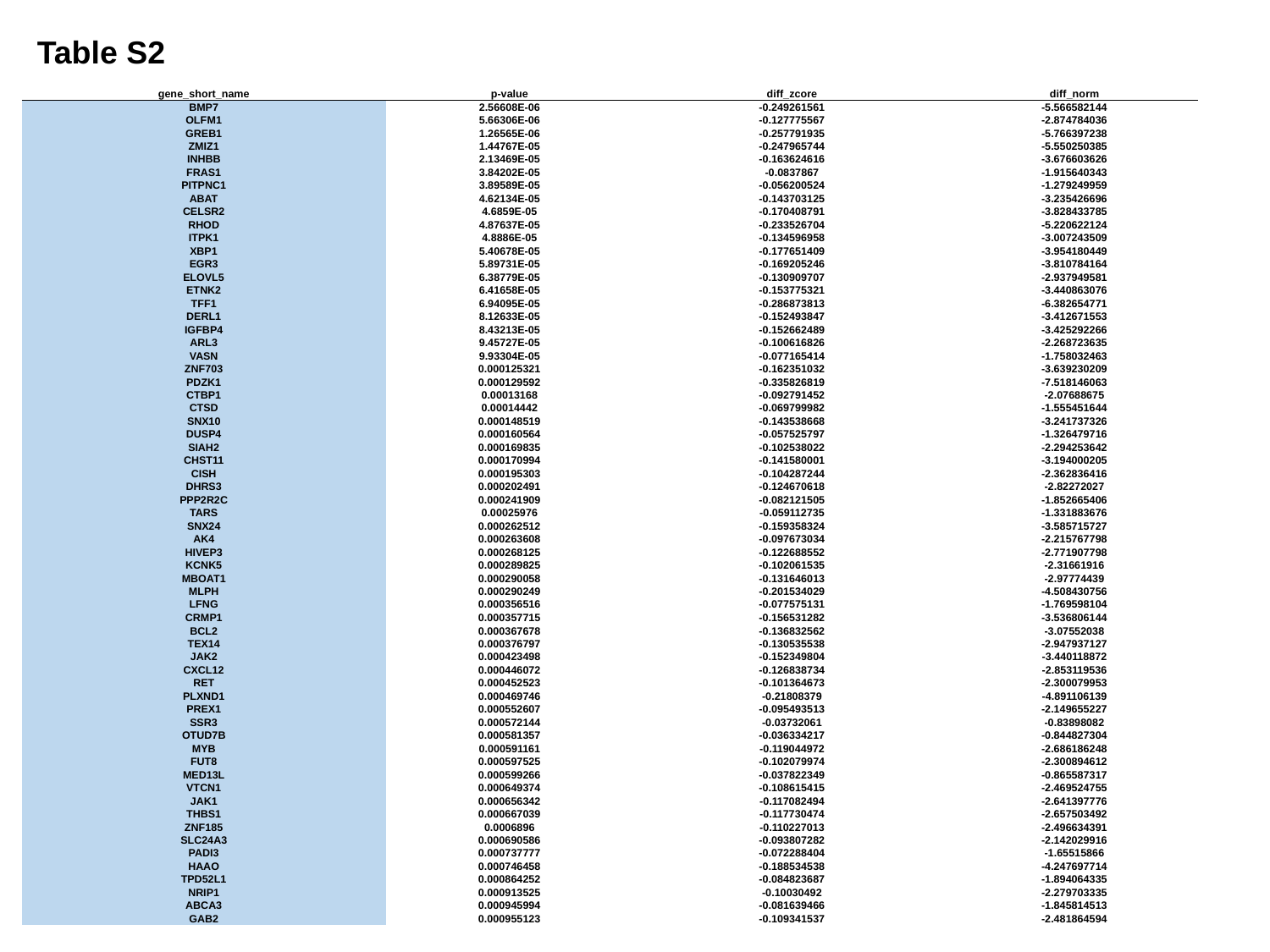

Table S2
| gene\_short\_name | p-value | diff\_zcore | diff\_norm |
| --- | --- | --- | --- |
| BMP7 | 2.56608E-06 | -0.249261561 | -5.566582144 |
| OLFM1 | 5.66306E-06 | -0.127775567 | -2.874784036 |
| GREB1 | 1.26565E-06 | -0.257791935 | -5.766397238 |
| ZMIZ1 | 1.44767E-05 | -0.247965744 | -5.550250385 |
| INHBB | 2.13469E-05 | -0.163624616 | -3.676603626 |
| FRAS1 | 3.84202E-05 | -0.0837867 | -1.915640343 |
| PITPNC1 | 3.89589E-05 | -0.056200524 | -1.279249959 |
| ABAT | 4.62134E-05 | -0.143703125 | -3.235426696 |
| CELSR2 | 4.6859E-05 | -0.170408791 | -3.828433785 |
| RHOD | 4.87637E-05 | -0.233526704 | -5.220622124 |
| ITPK1 | 4.8886E-05 | -0.134596958 | -3.007243509 |
| XBP1 | 5.40678E-05 | -0.177651409 | -3.954180449 |
| EGR3 | 5.89731E-05 | -0.169205246 | -3.810784164 |
| ELOVL5 | 6.38779E-05 | -0.130909707 | -2.937949581 |
| ETNK2 | 6.41658E-05 | -0.153775321 | -3.440863076 |
| TFF1 | 6.94095E-05 | -0.286873813 | -6.382654771 |
| DERL1 | 8.12633E-05 | -0.152493847 | -3.412671553 |
| IGFBP4 | 8.43213E-05 | -0.152662489 | -3.425292266 |
| ARL3 | 9.45727E-05 | -0.100616826 | -2.268723635 |
| VASN | 9.93304E-05 | -0.077165414 | -1.758032463 |
| ZNF703 | 0.000125321 | -0.162351032 | -3.639230209 |
| PDZK1 | 0.000129592 | -0.335826819 | -7.518146063 |
| CTBP1 | 0.00013168 | -0.092791452 | -2.07688675 |
| CTSD | 0.00014442 | -0.069799982 | -1.555451644 |
| SNX10 | 0.000148519 | -0.143538668 | -3.241737326 |
| DUSP4 | 0.000160564 | -0.057525797 | -1.326479716 |
| SIAH2 | 0.000169835 | -0.102538022 | -2.294253642 |
| CHST11 | 0.000170994 | -0.141580001 | -3.194000205 |
| CISH | 0.000195303 | -0.104287244 | -2.362836416 |
| DHRS3 | 0.000202491 | -0.124670618 | -2.82272027 |
| PPP2R2C | 0.000241909 | -0.082121505 | -1.852665406 |
| TARS | 0.00025976 | -0.059112735 | -1.331883676 |
| SNX24 | 0.000262512 | -0.159358324 | -3.585715727 |
| AK4 | 0.000263608 | -0.097673034 | -2.215767798 |
| HIVEP3 | 0.000268125 | -0.122688552 | -2.771907798 |
| KCNK5 | 0.000289825 | -0.102061535 | -2.31661916 |
| MBOAT1 | 0.000290058 | -0.131646013 | -2.97774439 |
| MLPH | 0.000290249 | -0.201534029 | -4.508430756 |
| LFNG | 0.000356516 | -0.077575131 | -1.769598104 |
| CRMP1 | 0.000357715 | -0.156531282 | -3.536806144 |
| BCL2 | 0.000367678 | -0.136832562 | -3.07552038 |
| TEX14 | 0.000376797 | -0.130535538 | -2.947937127 |
| JAK2 | 0.000423498 | -0.152349804 | -3.440118872 |
| CXCL12 | 0.000446072 | -0.126838734 | -2.853119536 |
| RET | 0.000452523 | -0.101364673 | -2.300079953 |
| PLXND1 | 0.000469746 | -0.21808379 | -4.891106139 |
| PREX1 | 0.000552607 | -0.095493513 | -2.149655227 |
| SSR3 | 0.000572144 | -0.03732061 | -0.83898082 |
| OTUD7B | 0.000581357 | -0.036334217 | -0.844827304 |
| MYB | 0.000591161 | -0.119044972 | -2.686186248 |
| FUT8 | 0.000597525 | -0.102079974 | -2.300894612 |
| MED13L | 0.000599266 | -0.037822349 | -0.865587317 |
| VTCN1 | 0.000649374 | -0.108615415 | -2.469524755 |
| JAK1 | 0.000656342 | -0.117082494 | -2.641397776 |
| THBS1 | 0.000667039 | -0.117730474 | -2.657503492 |
| ZNF185 | 0.0006896 | -0.110227013 | -2.496634391 |
| SLC24A3 | 0.000690586 | -0.093807282 | -2.142029916 |
| PADI3 | 0.000737777 | -0.072288404 | -1.65515866 |
| HAAO | 0.000746458 | -0.188534538 | -4.247697714 |
| TPD52L1 | 0.000864252 | -0.084823687 | -1.894064335 |
| NRIP1 | 0.000913525 | -0.10030492 | -2.279703335 |
| ABCA3 | 0.000945994 | -0.081639466 | -1.845814513 |
| GAB2 | 0.000955123 | -0.109341537 | -2.481864594 |
